# Supplementary material for: Potential decoupling of CO2 and Hg uptake process by global vegetation in the 21st century
Source: Nat Commun. 2024 May 27;15:4490. doi: 10.1038/s41467-024-48849-2 (PMC11130250; doi:10.1038/s41467-024-48849-2)
Supplement: Supplementary file 1 — Supporting Information [file 41467_2024_48849_MOESM1_ESM.pdf]

Supplementary information for

Potential decoupling of CO_2 and Hg uptake process by global vegetation in the 21st century

Tengfei Yuan[1], Shaojian Huang[1], Peng Zhang[1], Zhengcheng Song[1,2,3], Jun Ge[1,3], Xin Miao[1], Yujuan Wang[1], Qiaotong Pang[1], Dong Peng[1], Peipei Wu[1], Junjiong Shao[4], Peipei Zhang[5], Yabo Wang[6], Hongyan Guo[7], Weidong Guo[1], Yanxu Zhang[1,2,3*]

[1]School of Atmospheric Sciences, Nanjing University, Nanjing, Jiangsu 210023, China.

[2]Frontiers Science Center for Critical Earth Material Cycling, Nanjing University, Nanjing, Jiangsu 210023, China

[3]Joint International Research Laboratory of Atmospheric and Earth System Sciences, Nanjing University, Nanjing, Nanjing, Jiangsu 210023, China

[4]State Key Laboratory of Subtropical Silviculture, College of Forestry and Biotechnology, Zhejiang A&F University, Hangzhou, 311300, China

[5] CAS Key Laboratory of Mountain Ecological Restoration and Bioresource Utilization & Ecological Restoration and Biodiversity Conservation Key Laboratory of Sichuan Province, Chengdu Institute of Biology, Chinese Academy of Sciences, Chengdu, 610041, China

[6]College of Environmental Science and Engineering, Yangzhou University, Yangzhou 225127, China

[7]State Key Laboratory of Pollution Control and Resource Reuse, School of the Environment, Nanjing University, Nanjing, 210023, China

[*]Corresponding author. E-mail address: zhangyx@nju.edu.cn

Contents of this file

Supplementary Table 1 to 4

Supplementary Figures 1 to 17

Supplementary References

Supplementary Methods

CLM5-Hg

The nonstomatal uptake pathway entails the direct absorption of atmospheric Hg vapor via the cuticles in the epidermis of the canopy's upper layers[1] (Supplementary Fig. 4). We assume a 10% re-emission of Hg from foliage due to the release and subsequent reduction of previously sequestered Hg(0) within leaf tissue, along with a 15% re-emission from Hg deposited on leaf surfaces, transformed to Hg(0) through photoreduction[2–4].

Wet deposition of Hg(II) flux are speciated from the CAM6-Chem-Hg[5]. After being removed from the surfaces of leaves and soil, Hg(II) can penetrate the soil and has the ability to attach to reduced sulfur groups present in organic matter. Througfall flux are calculated as the sum of the Hg(II) dry deposition onto the canopy surface and the Hg(II) wet deposition that has not been reduced[6].

The below-ground process (soil pool) in CLM-Hg is divided into 25 layers, with the first 20 layers being the hydrological and biogeochemically active layers, and the last 5 layers being the parent rock layers. The litter is combined with soil C and nitrogen (N) components and is set to 20 layers. Each layer contains 4 litter pools: coarse woody debris pool (CWD), metabolic litter C pool (Litter 1), cellulose litter pool (Litter 2), and lignin litter pool (Litter 3), along with three soil pools: microbial pool (Soil 1), slow pool (Soil 2), and passive soil C and N pool (Soil 3).

The decomposition process of soil Hg is tied to the soil carbon pool, assuming that the Hg in the soil binds with soil carbon pools of different ages[6]. The transformation between different Hg pools and the microbial transformation rate are characterized using the conversion and respiration rates of the soil carbon pool in every layer[7]. Upon each transfer of carbon and Hg between pools, a fraction is lost—carbon as CO_2 respiration and Hg as Hg(0) evasion into the atmosphere. Following microbial decay, we estimate that 16% of atmospheric Hg(0) evades, with the remainder being reincorporated into organic matter[6,8]. In the framework of the single-level model structure, the foundational equation within decomposing Hg pools is as follows:

$$\frac{\partial C_i}{\partial t} = R_i + \sum_{j \neq 1} (1\text{-r}_j) T_{ji} K_j f_{Hg} C_j - K_i C_i \qquad (1)$$

Where C_i is the Hg content of pool i, R_i represent the Hg inputs from plant tissues directly to pool i (only non-zero for litter pools and CWD), k_i is the decay constant of carbon pool i; T_{ji} indicates the fraction of carbon directed from pool j to pool i, with a fraction r_j being lost as a respiration flux along the way. f_{Hg} is the constant representing the evasion into the atmosphere as Hg(0).

Incorporating the vertical dimension into the decomposition dynamics alters the balance equation as detailed below:

$$\frac{\partial C_i(z)}{\partial t} = R_i Z + \sum_{j \neq 1} (1\text{-r}_j) T_{ji} K_j(Z) f_{Hg} C_j(Z) - k_i(Z) + \frac{\partial}{\partial z}\left(D(z)\frac{\partial C_i}{\partial z}\right) + \frac{\partial}{\partial z}(A(Z) C_i) \qquad (2)$$

Where $C_i(z)$ is now defined at each model level in volumetric terms (gC m^{-3}), along with $R_i(z)$ and $k_j(z)$. Additionally, vertical transport is accounted for by the last two

terms, representing diffusive and advective transport, respectively. In the base model, advective transport is set to zero, leaving only a diffusive flux, with diffusivity D(z) defined for all decomposing carbon and Hg pools.

Our patterns are more realistic because a vertically resolved soil biogeochemistry scheme in CLM5 was introduced. This scheme features base decomposition rates that vary with depth and are modified by soil temperature, water, and oxygen limitations. It also includes vertical mixing of soil carbon and nitrogen due to bioturbation, cryoturbation, and diffusion[7].

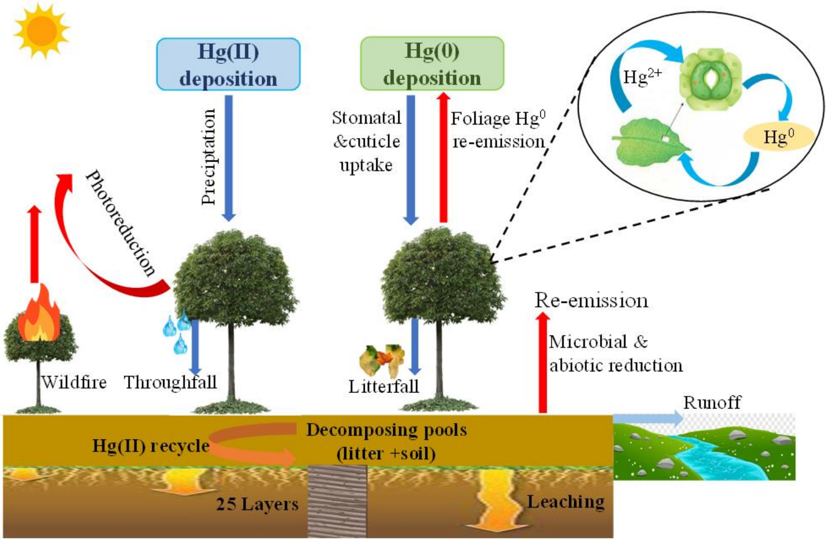

Supplementary Fig. 1 Terrestrial Hg cycle simulated by CLM5-Hg, as modified in Yuan et al.[12]. Blue arrows represent the deposition flux, and red arrows reperent the emission flux.

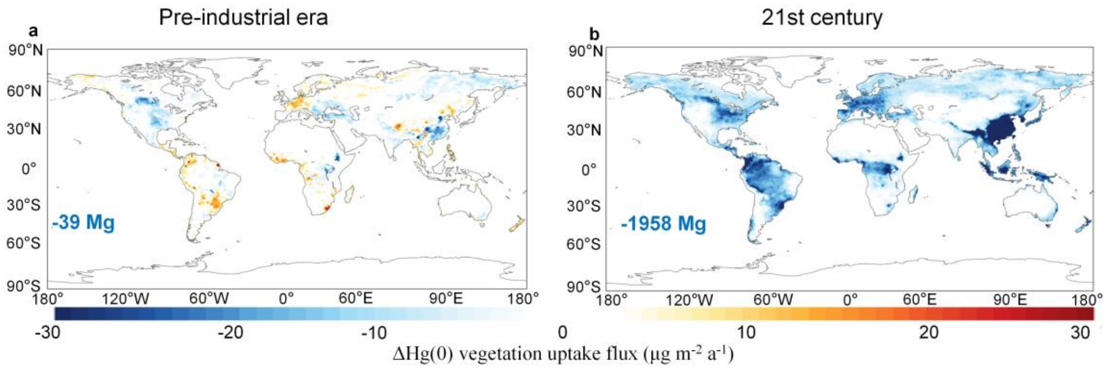

Supplementary Fig. 2 Modeled effects of climate change on global vegetation uptake of Hg(0) with combined all factors. a The numbers in the figure represent the total amount of global vegetation Hg(0) absorption changes between present-day and 1850. **b** The numbers in the figure represent the total amount of global vegetation Hg(0) absorption changes between present-day and 2100.

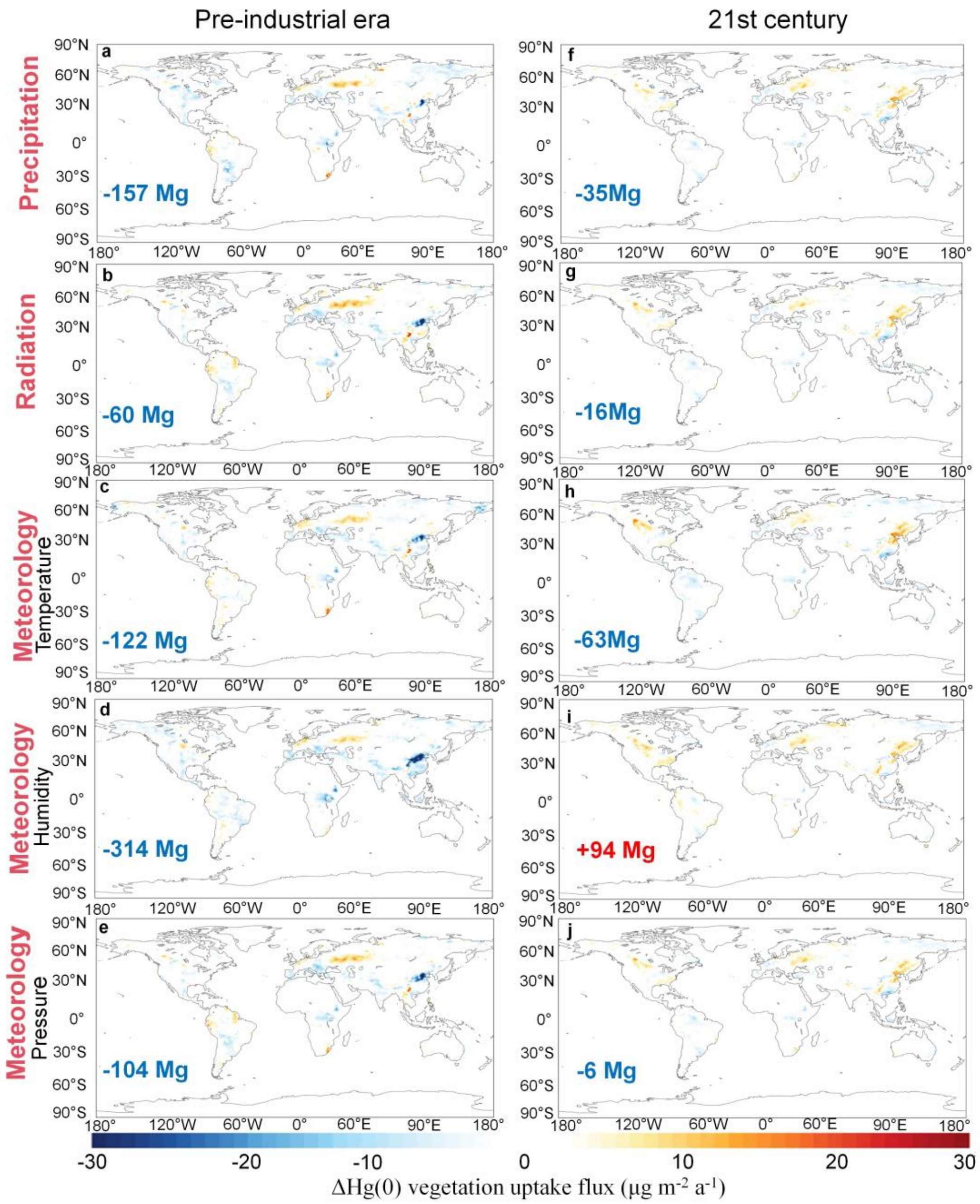

Supplementary Fig. 3 Modeled effects of climate change on global vegetation uptake of Hg(0) with individual factors excluding CO_2. a-e The numbers in the figure represent the total amount of global vegetation Hg(0) absorption changes between present-day and 1850. **f-j** The numbers in the figure represent the total amount of global vegetation Hg(0) absorption changes between present-day and 2100. Winds are not displayed because they show no significant changes.

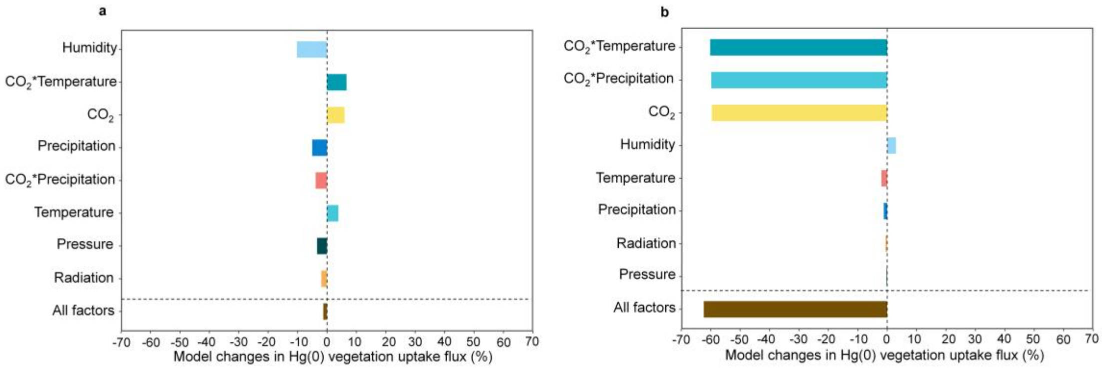

Supplementary Fig. 4 The modeled effect size of climate change factors. The percent changes of Hg(0) uptake by global vegetation on the land surface between present-day and 1850 (**a**), present-day and 2100 (**b**), contributed by individual factors and combined all factors.

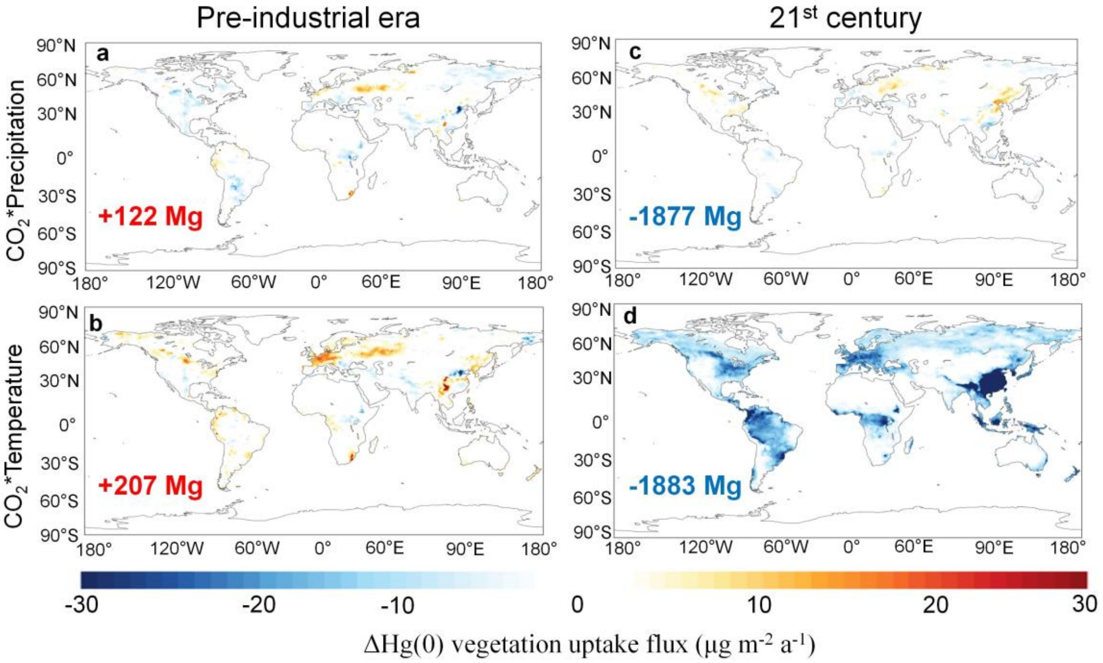

Supplementary Fig. 5 The effect of CO_2 interactions with temperature and precipitation on global vegetation uptake of Hg(0). a-b Changes in Hg(0) vegetation uptake flux caused by CO_2's interactions between present-day and 1850. The numbers in the figure represent the total amount of global vegetation Hg(0) absorption changes between present-day and 1850. **c-d** Changes in Hg(0) vegetation uptake flux caused by CO_2's interactions between present-day and 2100. The numbers in the figure represent the total amount of global vegetation Hg(0) absorption changes between present-day and 2100.

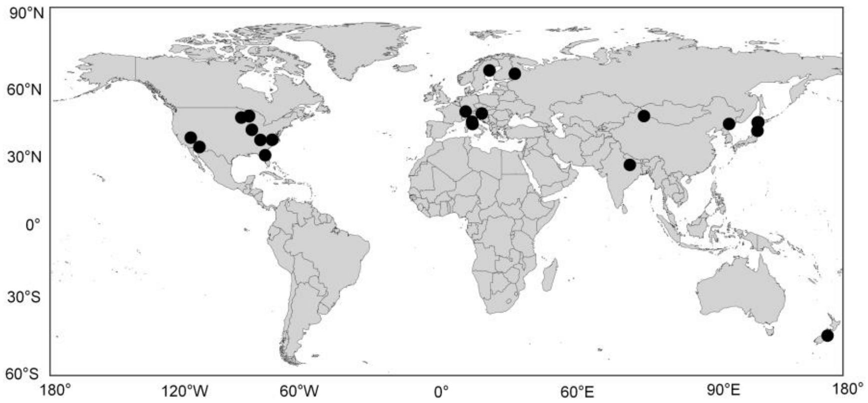

Supplementary Fig. 6 The global distribution of observational stomatal conductance (g_s) sites of manipulative eCO_2 experiment.

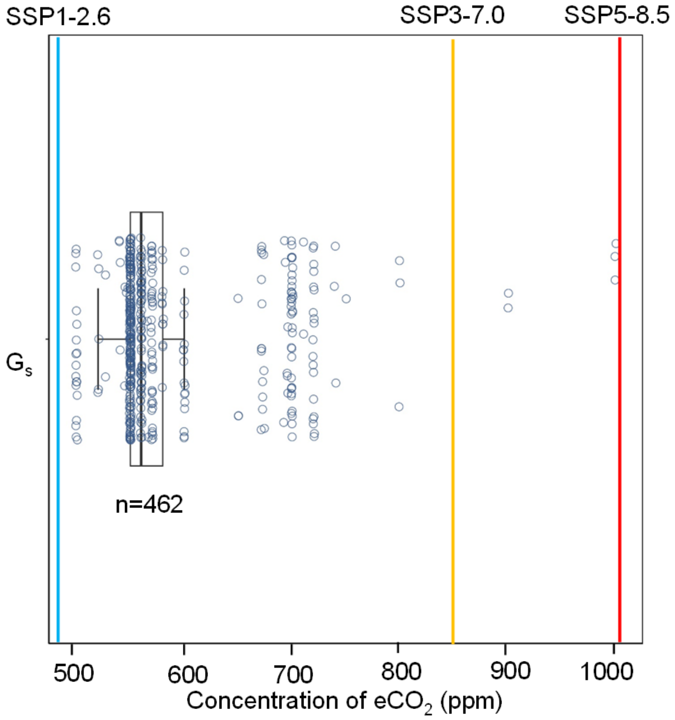

Supplementary Fig. 7 The CO_2 concentrations environment of g_s by manipulated elevated CO_2 (eCO_2) experiments and projected in Shared Socioeconomic Pathway (SSP) scenarios.

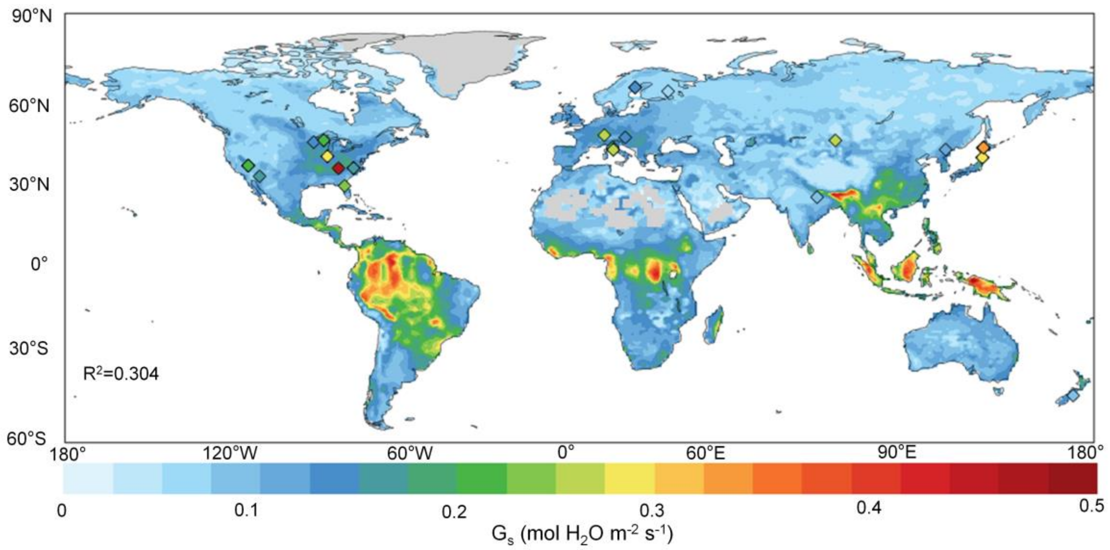

Supplementary Fig. 8 Stomal conductance modeld by CLM5-Hg at present-day. Observations (represented by rhombuses) are obtained from the global manipulative eCO_2 experiment.

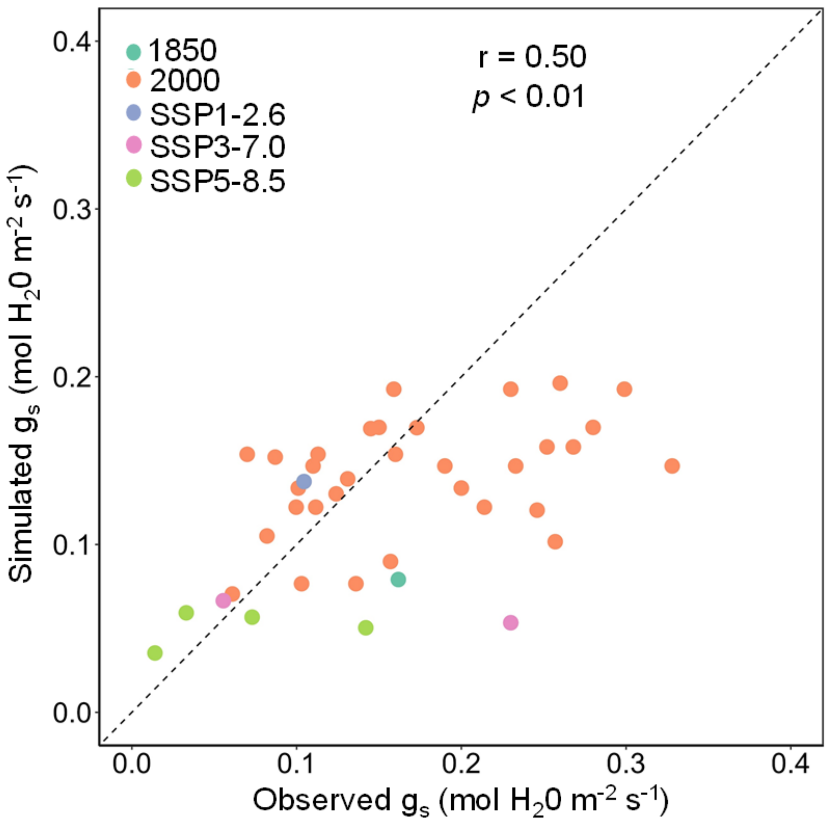

Supplementary Fig. 9 Comparison of simulated g_s (stomatal conductance) with observations under various CO_2 concentration levels: pre-industrial (1850), present-day, and projections under the Shared Socioeconomic Pathways (SSPs).

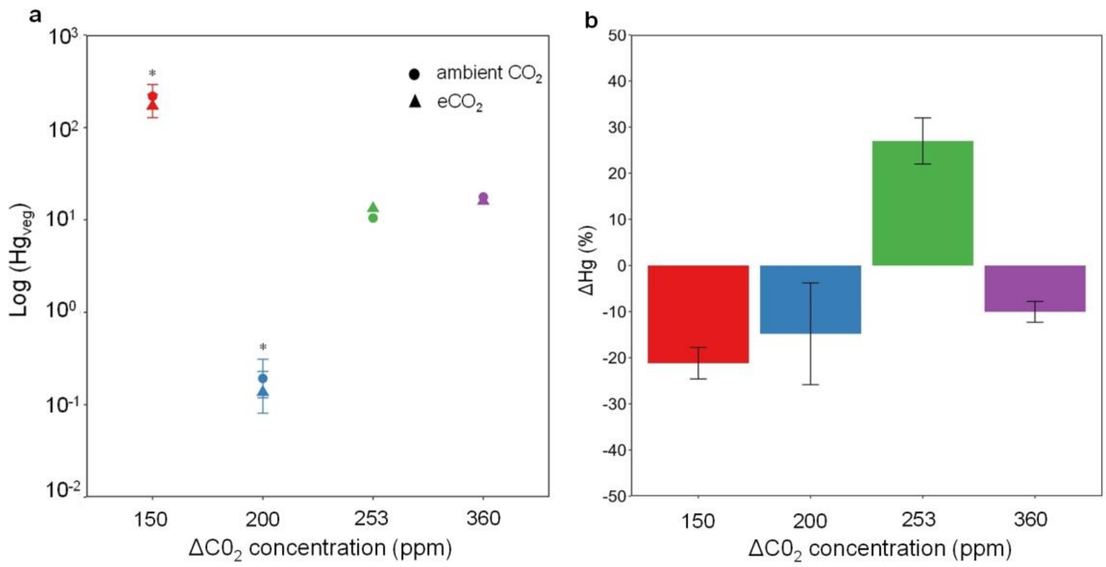

Supplementary Fig. 10 The effect of eCO_2 on vegetation Hg (Hg_{veg}). a, Concentration or flux of Hg in vegetation under different eCO_2 experimental conditions (n=70). 'Ambient CO_2' and 'eCO_2' represent the control and treatment conditions, respectively. Note: The y-axis is on a logarithmic scale. Two-way ANOVA, *$P < 0.05$. b, The percentage change in Hg flux or concentration in vegetation. Error bars represent the standard error.

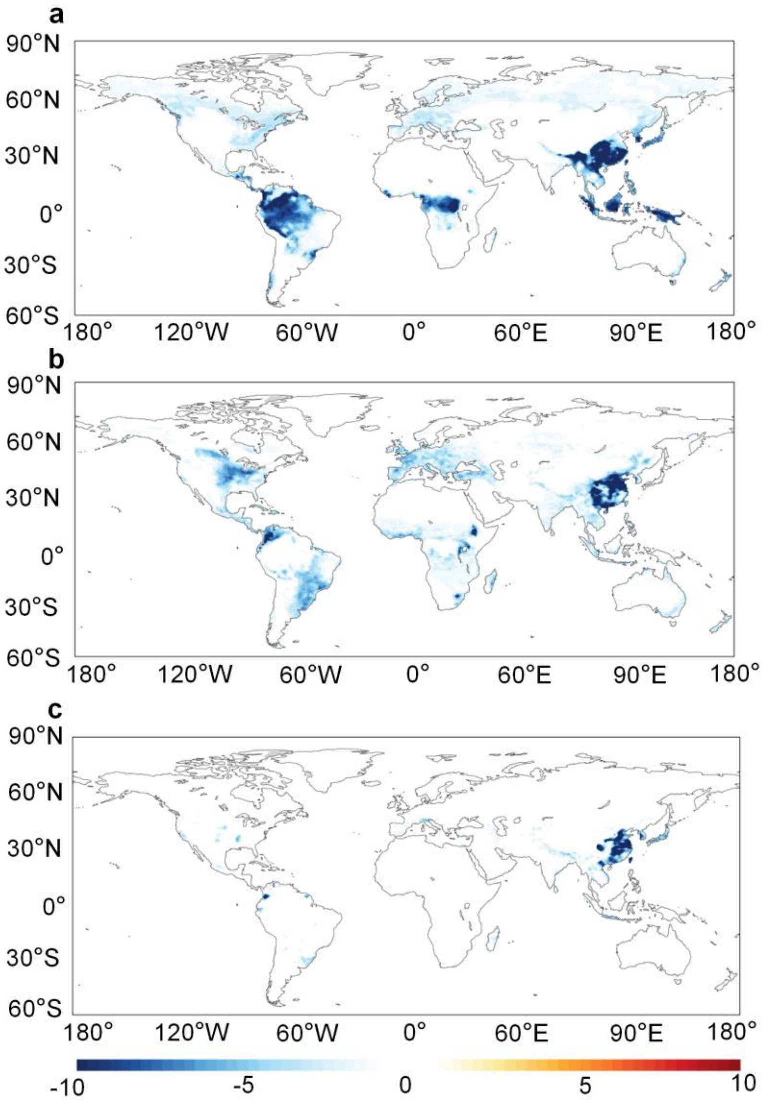

Supplementary Fig. 11 Change in global Hg(0) vegetation uptake caused by the eCO_2 between 2100 and the present-day in different vegetation types. a, trees, **b**, grasses, **c**, crops

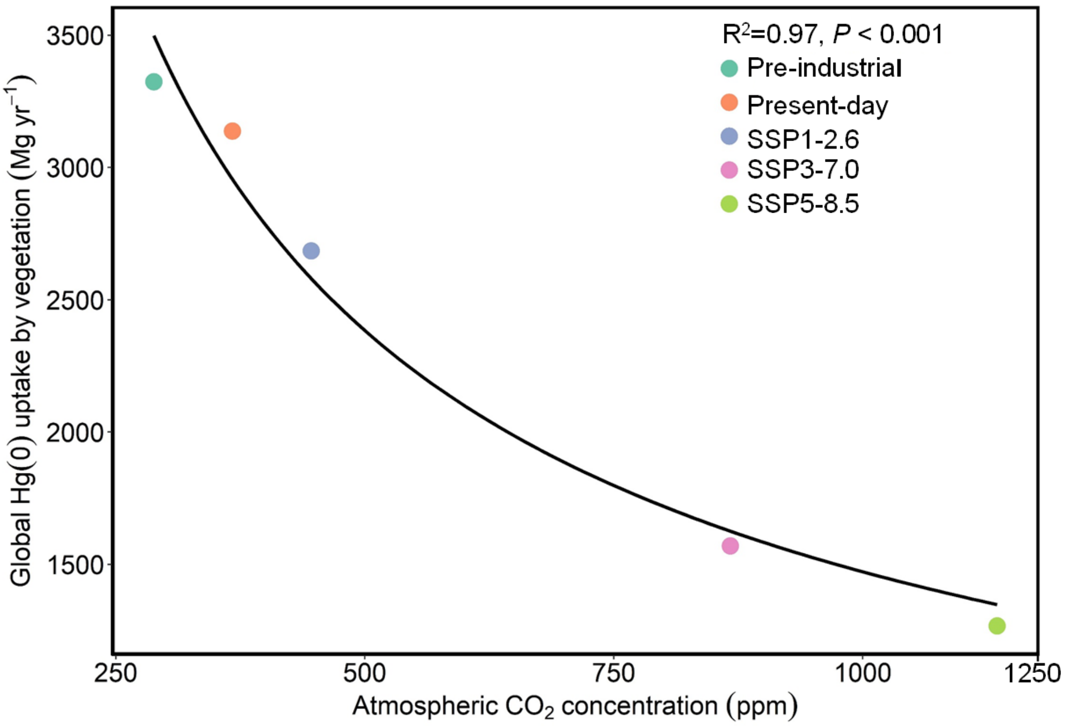

Supplementary Fig. 12 Relationship between atmospheric CO_2 concentration and global Hg(0) uptake by vegetation from historical emission scenario in 1850 to Shared Socioeconomic Pathways (SSP) emission scenarios in 2100.

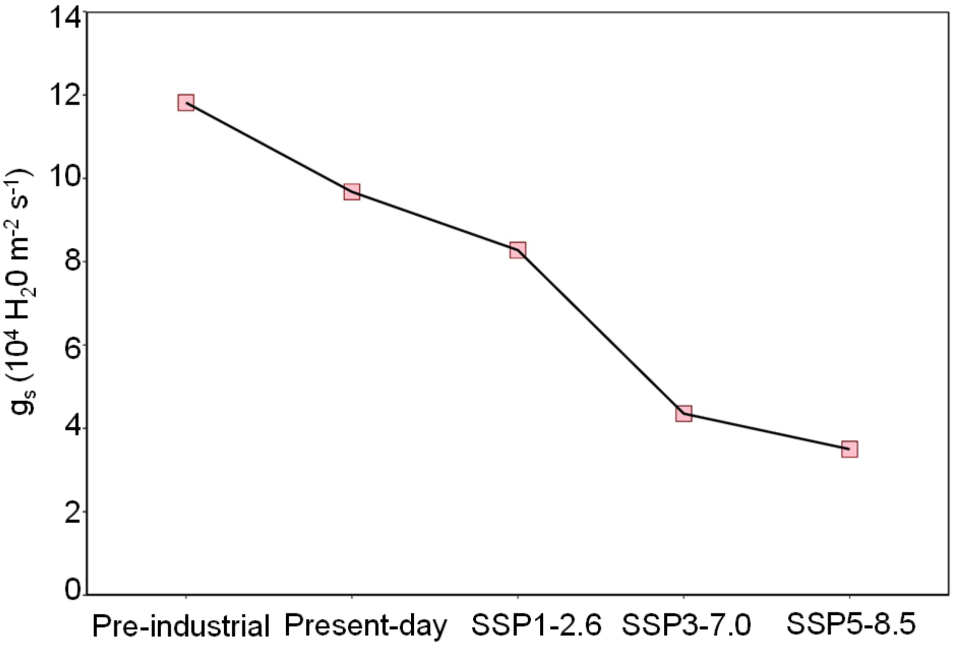

Supplementary Fig. 13 The trend of global average g_s from historical emission scenario in 1850 to Shared Socioeconomic Pathways (SSP) emission scenarios in 2100.

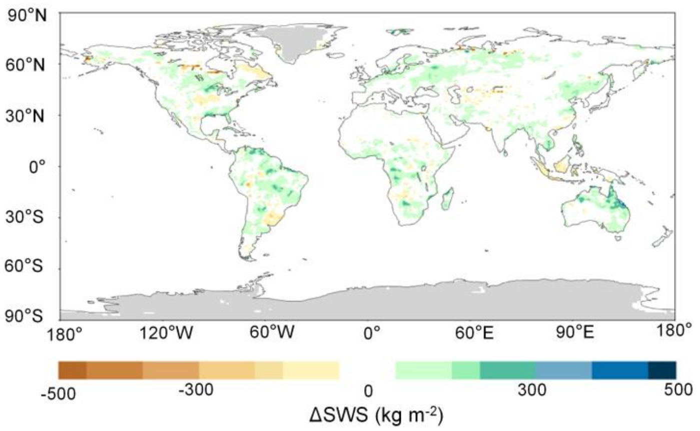

Supplementary Fig. 14 The effect of eCO_2 on global vegetation's water stress. Change in soil water storage (SWS) by the eCO_2 between 2100 and present-day.

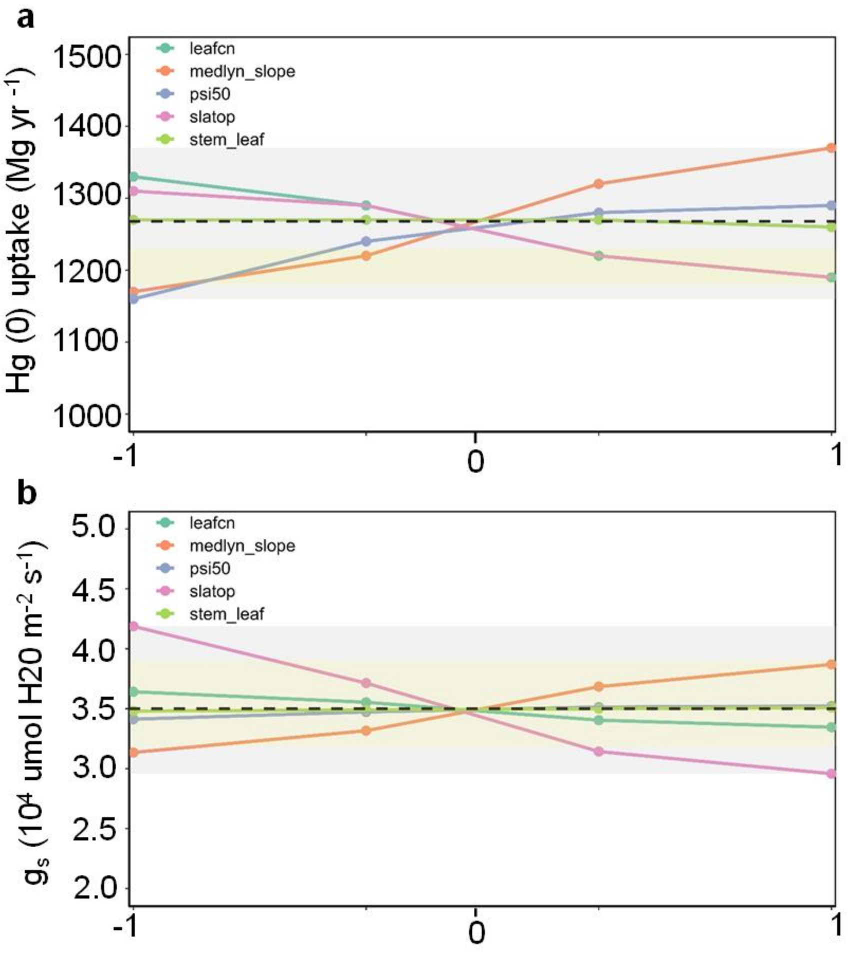

Supplementary Fig. 15 Response of model outputs to parameter perturbation across a −1 to +1 range of parameter variation (see Table S3), for (a) global vegetation Hg(0) uptake and (b) g_s. The grey and yellow shaded areas indicate the ranges of total uncertainty and uncertainty considering all parameters simultaneously, respectively. The black dotted line is the baseline, which indicates the original value.

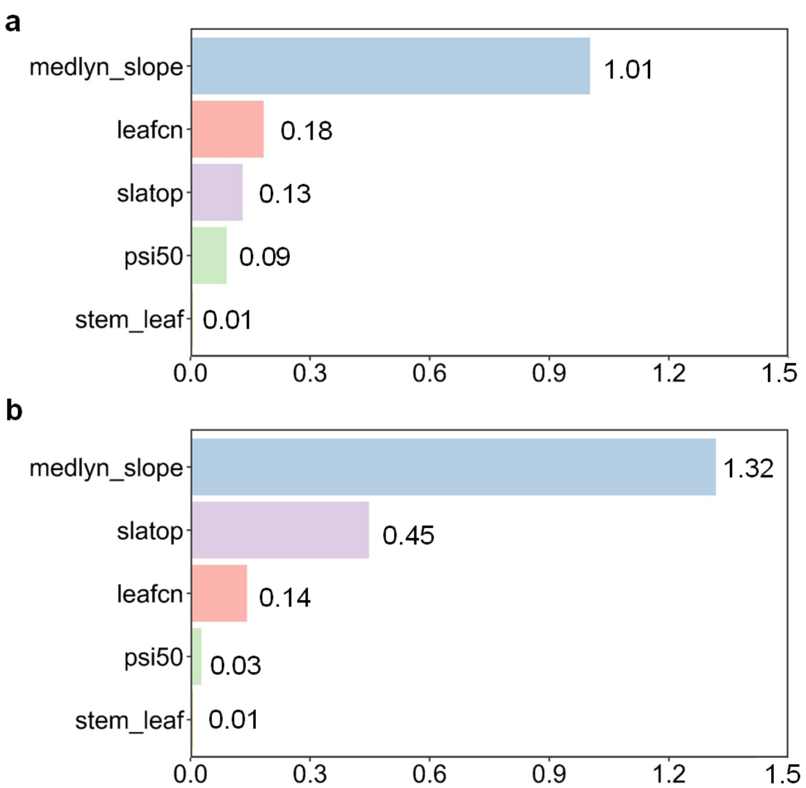

Supplementary Fig. 16 The coefficient of variation. a the relative uncertainty ranges of the global vegetation Hg(0) uptake. **b** the relative uncertainty range of (g_s)

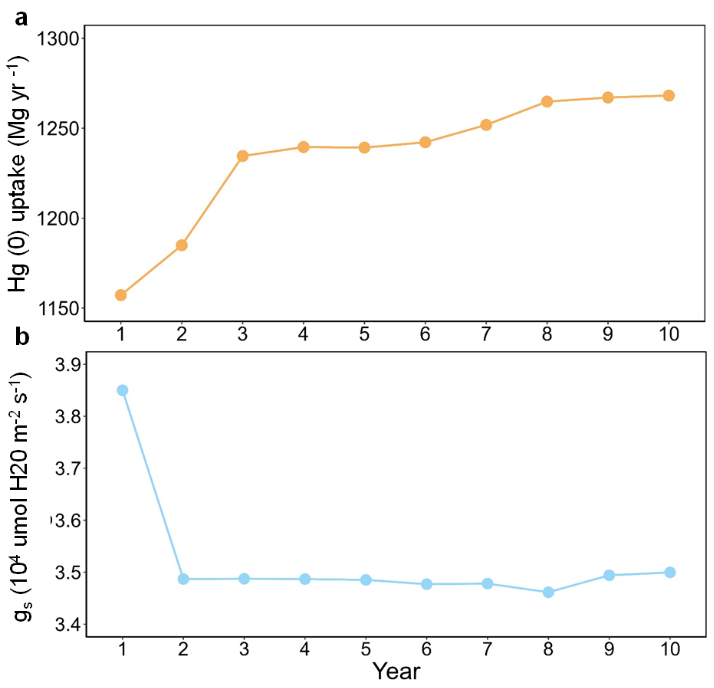

Supplementary Fig. 17 Time evolution of the change of CLM5-Hg model under the SSP5-8.5 scenario. a the change in global Hg(0) uptake by vegetation. **b** the change in global average g_s.

Supplementary Table 1 Experiment lists.

		Pre-industrial	Present-day	SSP1-2.6	SSP3-7.0	SSP5-8.5
CO_2		✓	baseline	✓	✓	✓
Pre.		✓	baseline			✓
Rad.		✓	baseline			✓
Meteorology	T.	✓	baseline			✓
	H.	✓	baseline			✓
	P.	✓	baseline			✓
	W.	✓	baseline			✓
All		✓	baseline			✓

Note. Pre:precipitation, Rad.:radiation, T.:Temperature, H: Humidity, P:Pressure, W: Wind, All: including all factor above.

Supplementary Table 2 The changes of Hg_{veg} (weighted mean response ratio) in response to eCO_2 in different vegetation types. CI, confidence interval; N, sampling size.

	Estimate (95% CI)	N	P-value	ΔCO_2 (ppm)
tree	-24.72%(-27.88% to -21.56%)	12	<0.001	200/360
grass	-11.75%(-16.11% to -7.39%)	110	<0.001	150/208
crop	-18.38%(-21.54% to 15.22%)	62	<0.001	253
all	-24.4%(-24.79% to -24.0%)	184	<0.001	/

Supplementary Table 3 The parameters, units and ranges (as Multipliers of the Original Values) of the uncertainty analysis. v1-v4 correspond to the multipliers use in the four level sensitivivity analysis runs (two higher and two lower than the default).

Parameter	Units	v1	v2	v3	v4
Medlyn_slope		-1_se	-0.5_se	+0.5_se	+1_se
slatop	m^2/gC	0.6761	0.8769	1.2785	1.4791
leaf_cn	gC/gN	0.7413	0.8932	1.1970	1.349
stem_leaf	gC/gC	0.5	0.75	1.25	1.5
psi50	mm	0.5	0.75	1.25	1.5

Supplementary Table 4 The uncertainty (coefficients of vatiation) of our results derived mainly from the parameter of medlyn model in CLM5 on global vegetation Hg(0) uptake and g_s**.**

**Item**	**Parameter**	**v1**	**v2**	**v3**	**v4**
**Hg(0)**	Medlyn_slope	1.006	1.182	0.396	1.425
	slatop	0.113	0.137	0.143	0.131
	leafcn	0.195	0.183	0.184	0.173
	psi50	0.168	0.103	0.051	0.039
	stem_leaf	0.007	0.007	0.006	0.006
**g_s**	Medlyn_slope	1.326	1.558	0.518	1.871
	slatop	0.606	0.497	0.366	0.323
	leafcn	0.156	0.145	0.138	0.126
	psi50	0.050	0.030	0.016	0.013
	stem_leaf	0.009	0.007	0.006	0.006

Supplementary References:

1. Wesely, M. L. Parameterization of surface resistances to gaseous dry deposition in regional-scale numerical models. *Atmospheric Environment (1967)* **23**, 1293–1304 (1989).
2. Yu, B. *et al.* Isotopic composition of atmospheric mercury in China: New evidence for sources and transformation processes in air and in vegetation. *Environmental Science and Technology* **50**, 9362–9369 (2016).
3. Demers, J. D., Blum, J. D. & Zak, D. R. Mercury isotopes in a forested ecosystem : Implications for air-surface exchange dynamics and the global mercury cycle. **27**, 222–238 (2013).
4. Yuan, W. *et al.* Stable Isotope Evidence Shows Re-emission of Elemental Mercury Vapor Occurring after Reductive Loss from Foliage. *Environmental Science & Technology* **53**, 651–660 (2019).
5. Zhang, P. & Zhang, Y. Earth system modeling of mercury using CESM2 – Part 1: Atmospheric model CAM6-Chem/Hg v1.0. *Geosci. Model Dev.* **15**, 3587–3601 (2022).
6. Smith-Downey, N. V, Sunderland, E. M. & Jacob, D. J. Anthropogenic impacts on global storage and emissions of mercury from terrestrial soils: Insights from a new global model. *Journal of Geophysical Research: Biogeosciences* **115**, (2010).
7. Lawrence, D. M. *et al.* The Community Land Model Version 5: Description of New Features, Benchmarking, and Impact of Forcing Uncertainty. *Journal of Advances in Modeling Earth Systems* **11**, 4245–4287 (2019).
8. Schaefer, K. *et al.* Potential impacts of mercury released from thawing permafrost. *Nature Communications* **11**, 4650 (2020).
9. Wang, X. *et al.* Emission-dominated gas exchange of elemental mercury vapor over natural surfaces in China. *Atmos. Chem. Phys.* **16**, 11125–11143 (2016).
10. Yuan, W. *et al.* Stable Mercury Isotope Transition during Postdepositional Decomposition of Biomass in a Forest Ecosystem over Five Centuries. *Environmental Science and Technology* **54**, 8739–8749 (2020).
11. Jiskra, M. *et al.* Mercury Deposition and Re-emission Pathways in Boreal Forest Soils Investigated with Hg Isotope Signatures. *Environmental Science & Technology* **49**, 7188–7196 (2015).
12. Yuan, T. *et al.* Buffering effect of global vegetation on the air-land exchange of mercury: Insights from a novel terrestrial mercury model based on CESM2-CLM5. *Environment International* **174**, 107904 (2023).
